# Oxygen-dependent histone lysine demethylase 4 restricts hepatitis B virus replication

**DOI:** 10.1016/j.jbc.2024.105724

**Published:** 2024-02-05

**Authors:** James M. Harris, Andrea Magri, Ana Rita Faria, Senko Tsukuda, Peter Balfe, Peter A.C. Wing, Jane A. McKeating

**Affiliations:** 1 Nuffield Department of Medicine, University of Oxford, Oxford, UK; 2 Chinese Academy of Medical Sciences Oxford Institute, University of Oxford, Oxford, UK

**Keywords:** hepatitis B virus, hypoxia, liver, histone lysine demethylase 4, viral hepatitis

## Abstract

Mammalian cells have evolved strategies to regulate gene expression when oxygen is limited. Hypoxia-inducible factors (HIF) are the major transcriptional regulators of host gene expression. We previously reported that HIFs bind and activate hepatitis B virus (HBV) DNA transcription under low oxygen conditions; however, the global cellular response to low oxygen is mediated by a family of oxygenases that work in concert with HIFs. Recent studies have identified a role for chromatin modifiers in sensing cellular oxygen and orchestrating transcriptional responses, but their role in the HBV life cycle is as yet undefined. We demonstrated that histone lysine demethylase 4 (KDM4) can restrict HBV, and pharmacological or oxygen-mediated inhibition of the demethylase increases viral RNAs derived from both episomal and integrated copies of the viral genome. Sequencing studies demonstrated that KDM4 is a major regulator of the hepatic transcriptome, which defines hepatocellular permissivity to HBV infection. We propose a model where HBV exploits cellular oxygen sensors to replicate and persist in the liver. Understanding oxygen-dependent pathways that regulate HBV infection will facilitate the development of physiologically relevant cell-based models that support efficient HBV replication.

Hepatitis B virus (HBV) is a global health problem with more than 290 million cases worldwide and over 880,000 deaths/year from liver diseases, including cirrhosis and hepatocellular carcinoma (HCC). HBV is an enveloped DNA virus and prototypic member of the *hepadnaviridae* that establishes its genome as an episomal, covalently closed circular DNA (cccDNA) in the nucleus of infected hepatocytes (reviewed in (1)). Current treatments for chronic hepatitis B include nucleos(t)ide analogues that suppress viral replication but are not curative, largely due to the persistence of the cccDNA (2) and failure to mount effective anti-viral immune responses (3, 4). In most cases, treatment is life-long and patients may still develop HCC (5), highlighting a clinical need for new curative therapies (6).

HBV replicates *via* the cccDNA genome with as few as 1 to 10 copies per cell (1) and is transcribed by RNA polymerase II to generate six major RNAs that include: pre-core (pC); pre-genomic (pgRNA); preS1, preS2 and S RNAs encoding the surface envelope glycoproteins and the X transcript for the multi-functional X protein (HBx) (7, 8). These viral RNAs are derived from four independent promoters that are regulated by host transcription factors and epigenetic modifiers. HBx promotes cccDNA transcription (9) by recruiting epigenetic modifiers, such as histone deacetylases (10), and antagonizing host repressors of gene transcription such as structural maintenance of chromosome 5/6 complex (11) and high mobility group box 1 (HMGB1) (12). Our understanding of the host factors and their interplay with HBx to regulate cccDNA transcription is limited.

Virus replication is dependent on the cellular microenvironment, which is fundamentally shaped by local oxygen tension. Single-cell RNA sequencing of the human and murine liver showed an association between hypoxic signaling pathways and the hepatocellular transcriptome and metabolome (13, 14). The hypoxia-inducible factor (HIF) signaling axis drives the immediate hypoxic response, where oxygen-dependent prolyl-hydroxylase (PHD) enzymes hydroxylate HIF-α subunits resulting in proteasomal degradation. Hypoxic inactivation of the PHDs leads to stable expression of HIF-α and dimerization with HIF-1β that activates transcription of a plethora of genes involved in energy metabolism (15). We previously reported that HIFs activate cccDNA transcription by binding to conserved hypoxic response elements (HRE) in the viral genome (16). Additional studies report a role for HIFs to promote the abundance of HBV RNA and core antigen (17) and to suppress cccDNA deamination by Apolipoprotein B mRNA Editing Catalytic Polypeptide-like 3 B (APOBEC3B) (18). HIFs have been reported to influence the replication of diverse viral pathogens, enhancing Epstein Barr (19, 20) and Kaposi's sarcoma-associated herpes viruses (21), while suppressing Influenza, HIV-1, Respiratory Syncytial Virus and SARS-CoV-2 replication (22, 23, 24, 25). These contrasting effects may reflect different replicative mechanisms of DNA and RNA viruses, along with the variable oxygen tension at the sites of virus replication (26).

While HIFs regulate the transcriptional response to hypoxia, the cellular response to low oxygen extends beyond this signaling axis, involving the 2-oxoglutarate-dependent dioxygenases (2-OGDDs). This family of oxygen-dependent enzymes regulates gene expression at many levels, including nucleic acid methylation and histone modification (27); however, their role in the replication of HBV or other viral pathogens is not understood. Histone lysine demethylases (KDMs) are the largest sub-family of 2-OGDDs and comprise eight isoforms. These enzymes catalyze the demethylation (me3 > me2; me2 > me1) of specific lysine residues on histones 3 and 4 that can activate or repress gene expression (27). KDM5 and 6 were identified as oxygen-sensors that regulate gene expression independent of HIF signaling (28, 29). Under hypoxic conditions, KDM activity is inhibited, resulting in the accumulation of specific histone methylation marks that modify chromatin conformation and orchestrate the transcriptional response to hypoxia (30). Our study uncovers a role for KDM4 in regulating hepatocellular gene expression, where pharmacological or oxygen-mediated inhibition of the demethylase promotes HBV infection and activity of episomal and integrated viral genomes.

## Results

### KDM4 regulates HBV RNA levels in an oxygen-dependent manner

**Figure 1:**
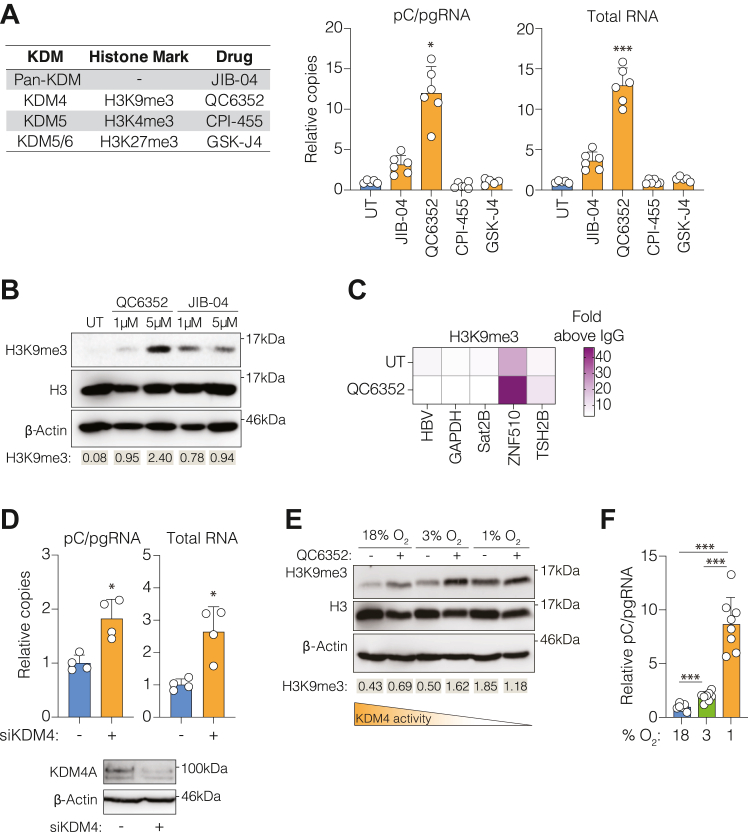
**KDM4 regulates the abundance of HBV RNAs in an oxygen-dependent manner.***A*, HepG2-NTCP cells were infected with HBV (genotype D strain, ayw) and treated with inhibitors targeting all KDMs (JIB-04: 1 μM), KDM4 (QC6352: 5 μM), KDM5 (CPI-455: 1 μM) or KDM5/6 (GSK-J4: 2 μM). After 72 h cellular RNAs were extracted and HBV transcripts (pC/pgRNA and total RNA) were assessed by qPCR, with copies expressed relative to untreated (UT) cells. *B*, HepG2-NTCP cells were treated with QC6352 or JIB-04 (1 and 5 μM) and H3K9 trimethylation or total H3 protein levels detected by Western blot. Densitometric analysis of H3K9 trimethylation relative to β-Actin and the mean value from two independent experiments are shown. *C*, HepAD38 cells were cultured in the presence of QC6352 (5 μM) for 72 h. Cells were fixed and chromatin extracted before immunoprecipitation with antibodies specific to H3K9me3 or an irrelevant IgG control. RT-qPCR was used to amplify either HBV DNA (38) or host gene loci, and percentage of input calculated. Data are expressed as fold above the IgG control in each condition, for each primer pair, and are derived from two independent experiments (38). *D*, HepG2-NTCP cells were transfected with siRNAs targeting KDM4A-D, infected with HBV, and cultured for 72 h. Cellular protein and RNA were extracted: pC/pgRNA and total HBV RNA were assessed by qPCR and KDM4A expression was assessed by Western blot. *E*, HepG2-NTCP cells were cultured at 18%, 3% or 1% O_2_ in the presence or absence of QC6352 (5 μM) for 72 h. Cells were lysed and H3K9me3, histone (H3), and β-Actin were detected by Western blot. Densitometric analysis of H3K9 trimethylation relative to β-Actin and the mean value from two independent experiments are shown. Cartoon indicates the relationship between oxygen levels and KDM4 activity. *F*, HepG2-NTCP cells were infected with HBV as described above, cultured at 18%, 3% or 1% O_2_ for 72 h and pC/pgRNA levels quantified and expressed relative to infected cells maintained at 18% oxygen. Data are presented as mean ± SD from (*A*) n = 6, (*D*) n = 4, and (*F*) n = 8, with significance determined using Mann–Whitney *U* tests, with Bonferroni corrections for multiple comparisons (ns = *p* > 0.05, ∗*p* < 0.05, ∗∗∗*p* < 0.001). See also Figs. S1–S3.

As KDM5 and 6 can regulate the cellular transcriptional response to low oxygen (28, 29), we investigated their role in regulating the abundance of HBV transcripts by treating infected HepG2 cells with the pan-KDM inhibitor JIB-04 (31), or with compounds targeting KDM5 (32) and KDM6 (33). In our previous study, we optimized a 72 h infection protocol, where dimethyl sulfoxide (DMSO) was not used to arrest the cell cycle as we previously reported this agent blunted the cellular hypoxic response (16). Therefore, we adopted the same experimental model here and detected HBV transcripts by qPCR using primers targeting a unique region in the 5′ regions of the genome (pC/pgRNA) or the 3′ terminus shared by all transcripts (total RNA) (34). JIB-04 induced a modest increase in pC/pgRNA levels, whereas CPI-455 and GSK-J4, inhibitors of KDM5 and KDM6 respectively, had no discernible effect on the level of viral RNAs (Fig. 1*A*), while regulating the methylation of their respective histone targets (Fig. S1*A*). Phylogenetic analysis identified homology between KDM4 and 5 (35), and we expanded our analysis to include KDM4 (36). We observed a striking increase in the levels of pC/pgRNA and total HBV RNAs after inhibiting KDM4 with QC6352, accompanied by an increase in the levels of H3K9 trimethylation and limited evidence of cytotoxicity (Figs. 1*B* and S1*B*). To assess the effect of QC6352 treatment on viral and host-associated H3K9-trimethylation we used HepAD38 cells (37) that stably express integrated HBV DNA with QC6352 for 72 h, extracted chromatin and immuno-precipitated cell lysates with antibodies against H3K9me3 (KDM4-regulated), H3K4me3 (KDM5-regulated), and H3K27me3 (KDM6-regulated). Amplifying HBV DNA using primers spanning the nucleosome-associated region (38) showed modest evidence of repressive H3K9me3 on the viral genome, consistent with previous reports (38), and a negligible effect of QC6352 treatment (Fig. 1*C*). Zinc Finger Protein 510 (ZNF510) has been reported to be enriched for H3K9me3 (39), we confirmed this in our model system and QC6352 increased H3K9me3 on the ZNF510 gene locus as expected (Fig. 1*C*). We did not detect any H3K9me3 on the GAPDH promoter, an actively transcribed gene, and minimal association with reported H3K4me3 and H3K27me3 control genes TSH2B and Sat2B (Fig. S1*C*), respectively. Importantly, we detected both H3K4- and H3K27 trimethylation associated with HBV DNA and their host gene loci (GAPDH, Sat2B, and TSH2B, respectively (39, 40, 41)), with modest changes following QC6352 treatment, consistent with KDM4 specific inhibition (Fig. S1*C*).

The KDM4 family consists of five isoforms (A-E), and we assessed their expression in hepatocytes and hepatoma lines using published RNA-seq data (42, 43). KDM4A was the most abundantly expressed mRNA and KDM4B-D transcripts were detected at lower levels. Notably, KDM4E was not detected which may reflect low transcript frequency (Fig. S1*D*) (44). We noted similar KDM4A-D gene expression in all hepatoma lines and primary hepatocytes, suggesting that HepG2 cells provide a representative model to study KDM4-regulated pathways. We sought to confirm our earlier findings by genetic silencing of KDM4. As earlier published studies reported compensatory mechanisms when silencing a single KDM4 isoform (45, 46), we opted to deliver a pool of siRNAs targeting KDM4A-D into HepG2-NTCP cells. Silencing was confirmed by measuring KDM4A-D transcripts (Fig. S1*E*) and KDM4A expression by western blotting (Fig. 1*D*), as antibodies against other KDM4 isoforms were unavailable. We observed a significant increase in pC/pgRNA and total RNA levels (Fig. 1*D*).

As pharmacological inhibition and siRNA knockdown of KDM4 increased the abundance of HBV transcripts, we assessed the impact of manipulating cellular oxygen levels on KDM4 activity. Since KDM4 enzymatic activity is oxygen-dependent (47), we hypothesized that its ability to demethylate H3K9me3 would be diminished under hypoxic conditions. HepG2-NTCP cells were cultured at 18%, 3% or 1% oxygen in the presence or absence of QC6352 and KDM4 activity was assessed. There was a negligible change in H3K9 trimethylation between 18% and 3% oxygen; however, its abundance increased 3-fold at 1% oxygen, consistent with reduced KDM4 activity (Fig. 1*E*). QC6352-treated cells showed increased H3K9me3 levels at 18% and 3% oxygen; however, the drug caused no further changes in methylation at 1% oxygen (Fig. 1*E*). We confirmed the cellular response to hypoxia by assessing HIF-regulated gene expression (Fig. S2*A*) and found that QC6352 treatment did not alter the basal expression of canonical HIF target genes (Fig. S2*B*). Culturing HBV-infected HepG2 cells at 18%, 3% and 1% oxygen showed a modest but significant increase in pC/pgRNA levels at 3% oxygen and a 10-fold induction at 1% oxygen (Fig. 1*F*). As KDM4 activity was reduced at 1% oxygen, we predicted that QC6352 treatment under these conditions would have minimal pro-viral activity. Indeed, treating infected cells cultured at 1% oxygen had a negligible effect on the level of pC/pgRNAs compared to an 8-fold increase in cells propagated at 18% oxygen (Fig. S3). Collectively, these data demonstrate that inhibition of KDM4 potentiates the steady-state level of HBV transcripts.

### Hypoxic regulation of HBV transcripts involves HIFs and KDM4

**Figure 2:**
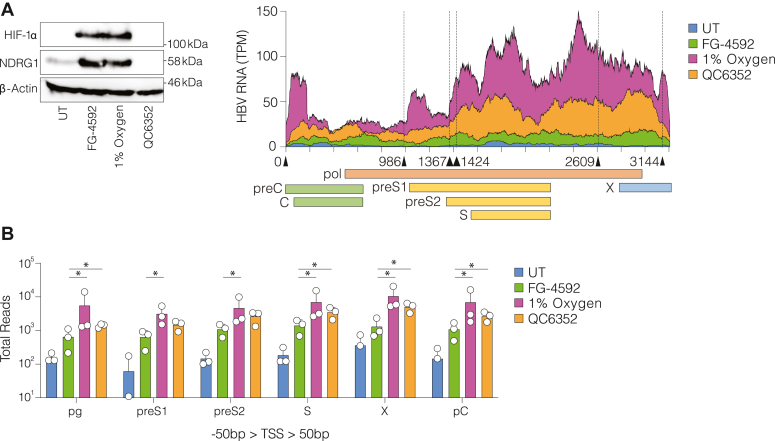
**Hypoxicregulation of HBV transcripts involves KDM4 and HIFs.***A*, HepG2-NTCP cells were infected with HBV and cultured under 1% oxygen or treated with FG-4592 (30 μM) or QC6352 (5 μM) for 72 h. HIF-1α and NDRG1 protein expression levels were assessed using western blotting. Total cellular RNAs were sequenced by Illumina and the viral reads mapped to the HBV genome, with the start coordinate at the pgRNA TSS (nt1816). Sequence coverage (number of transcripts/million reads - TPM) was averaged from n = 3 independent biological replicates from UT samples, FG-4592, 1% oxygen and QC6352 and plotted along the 3.2 kb viral genome. The schematic cartoon depicts the location of HBV protein-coding regions, with x-axis ticks denoting 200 bp intervals. *B*, viral reads around the TSS ± 50 bp for the major viral RNAs were quantified and differences were evaluated using multiple Mann–Whitney *U* tests with Holm-Sidak *p*-value corrections, where all treated samples were greater than the UT control (∗*p* < 0.05). Sequencing data are derived from n = 3 biological samples per condition.

We previously reported that HIFs positively regulate HBV transcription by binding to cccDNA (16). Our new data provides an additional dimension to this model in which hypoxia can inhibit KDM4 to foster a permissive environment for HBV replication. To explore the effects of QC6352 on the viral transcriptome, we performed Illumina RNA-sequencing on HBV-infected HepG2-NTCP cells cultured at 1% oxygen or treated with either QC6352 or the PHD inhibitor FG-4592. We confirmed HIF-1α expression and induction of N-Myc Downstream Regulated Gene 1 (NDRG1; a validated HIF target gene) in response to FG-4592 or 1% oxygen conditions, but not in response to QC6352 treatment (Fig. 2*A*). Aligning sequence reads to the HBV genome showed that FG-4592, QC6352 and 1% oxygen resulted in higher transcripts per million (TPM) (7.25-, 12.34-, and 13.42-fold respectively) compared to the untreated samples, with the highest viral RNA levels noted under 1% oxygen conditions (Fig. 2*A*). Our data are comparable with both Lim *et al*. (7) and published PacBio long-read sequencing data (48), showing major peaks in reads spanning the preS1-S region and the conserved 3′ end of the viral genome. Despite the caveats of transcript fragmentation in Illumina sequencing, we assessed the coverage depth at each of the major transcriptional start sites (TSS) ± 50 bp (7). These analyses showed that 1% oxygen and QC6352 induced significantly more viral reads than FG-4592 across all TSS (Fig. 2*B*), consistent with an increase in the major viral RNAs. Taken together, these data suggest that while HIFs promote viral replication, they are not solely responsible for the hypoxic potentiation of viral replication, and that KDM4 acts as an oxygen-dependent repressor of HBV transcription.

### KDM4 inhibition activated HBV episomes and integrants

**Figure 3:**
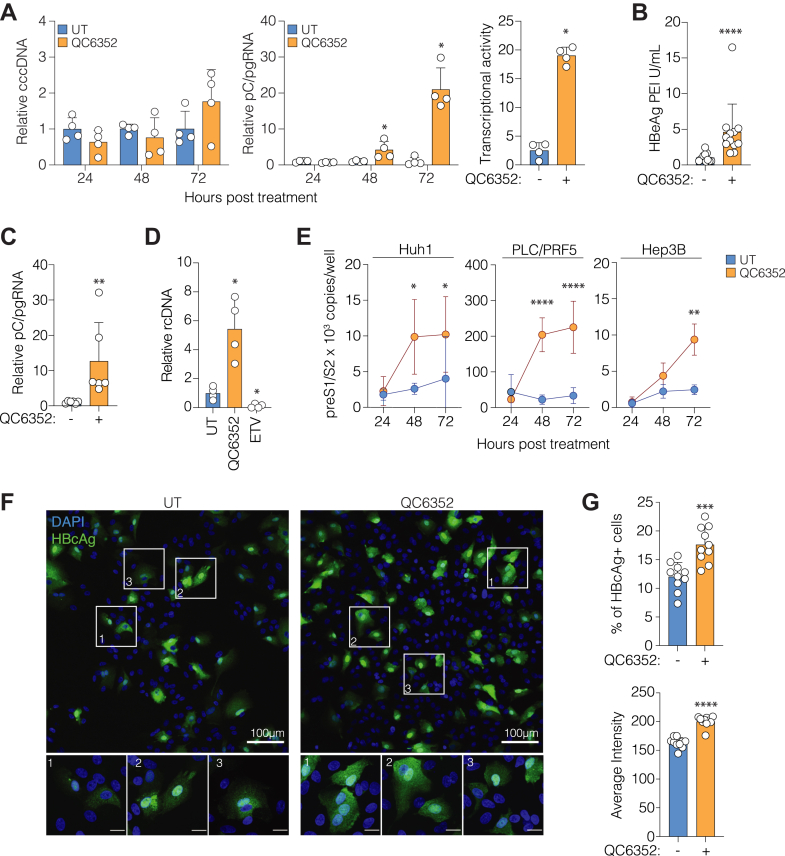
**KDM4 inhibition activates HBV episomes and integrants.***A*, quantification of cccDNA and pC/pgRNA in HBV-infected HepG2-NTCP cells treated with QC6352 (5 μM) sampled at 24, 48, and 72 h post-infection. cccDNA and pC/pgRNA were quantified by qPCR, and copies expressed relative to the Prion Protein (PRP) gene or β-Actin, respectively. At each time point, the data are expressed relative to UT cells. *B*, transcriptional activity at 72 h post-infection, defined as pC/pgRNA relative to cccDNA. Extracellular HBeAg was measured from QC6352 (5 μM) treated HBV-infected HepG2-NTCP cells at 6 days post-infection. *C*, HepG2 cells harbouring episomal HBV genomes (HepG2-pEpi) were treated with QC6352 (5 μM) for 72 h and pC/pgRNA levels were quantified and expressed relative to UT cells. *D*, HepAD38 cells were treated with either QC6352 (5 μM) or the reverse-transcriptase inhibitor Entecavir (1 μM) for 72 h, and extracellular encapsidated rcDNA detected in the supernatant. *E*, hepatoma cell lines harbouring integrated copies of the HBV genome were treated with QC6352 (5 μM), RNA extracted at 24 h intervals and intracellular preS1/2 mRNAs measured by qRT-PCR. *F*, HepG2-NTCP cells were differentiated for 72 h in 2.5% DMSO before infection with HBV (MOI 1000) for 10 days in the presence or absence of QC6352 (5 μM). Drugs were replenished every 3 days, and cultures were fixed and co-stained for HBcAg (Dako) and DAPI. Representative images at 20× and 100× (scale bar = 20 μm) magnification are shown. *F*, images were analyzed for the frequency of infected cells and for the intensity of HBcAg staining. Quantification was based on at least 3500 cells, from ten independent fields of view and significance was assessed using Mann–Whitney *U* tests. Data are presented as mean ± SD from (*A*) n = 4 (*B*) n = 4 to 12 and (*C* and *E*) n = 6 (*D*) n = 4 (*G*) n = 10 replicates and differences assessed by (*A*, *B*, *C*, *D*, and *G*) Mann-Whitney U tests or (*E*) 2-way ANOVA; (∗*p* < 0.05, ∗∗*p* < 0.01, ∗∗∗*p* < 0.001, ∗∗∗∗*p* < 0.0001). See Fig. S4.

To assess whether QC6352 increases the level of HBV transcripts by regulating the copies of viral DNA, we measured cccDNA and pC/pgRNA levels over 72 h. We used a recently published qPCR method to quantify cccDNA, which includes a rigorous exonuclease digestion to remove rcDNA before amplification and interpolated cccDNA copies from a standard curve (49). Comparable levels of cccDNA were measured at all time points; however, we observed a modest but significant increase in pC/pgRNA after 48 h, which increased 20-fold by 72 h (Fig. 3*A*). Expressing pC/pgRNA relative to cccDNA suggests that QC6352 enhances cccDNA transcriptional activity, rather than modulating the abundance of the transcriptional template (Fig. 3*B*). pC RNA encodes hepatitis B e antigen (HBeAg) and we show a concomitant increase in secreted antigen levels following QC6352 treatment (Fig. 3*B*). To extend our observations, we assessed the role of KDM4 in HepG2-pEpi cells, which carry an episomal cccDNA-like template that is resistant to integration (16), and observed a significant increase in pC/pgRNA levels following QC6352 treatment (Fig. 3*C*). To assess whether the increased levels of intracellular pgRNA are reflected in a greater secretion of encapsidated viral genomes we selected to use HepAD38 cells that support robust virion production (37). HepAD38 cells were treated with QC6352 or the reverse transcriptase inhibitor entecavir for 72 h. We observed an increase in intracellular pC/pgRNA in response to QC6352 treatment (Fig. S4) and a concomitant increase in particle release (Fig. 3*D*). As expected, entecavir reduced the level of extracellular encapsidated genomes (Fig. 3*D*).

During infection, aberrant HBV replication can generate double-stranded linear DNA (dslDNA) that can integrate into the host chromosome (reviewed in (50)). These integrated copies do not produce infectious viruses due to the disruption of the pC/pg RNA reading frame, yet can still generate preS1/S2, S and X RNAs. QC6352 treatment of hepatoma cell lines that carry integrated HBV genomes (Huh-1, PLC/PRF5 and Hep3B cells (51)) induced a time-dependent increase in the level of preS1/2 RNAs (Fig. 3*E*). Taken together, these data suggest that KDM4 regulates both integrated and episomal forms of the viral genome.

The HBV core is a structural component of the capsid and is frequently used to identify infected hepatocytes, in both *in vitro* and *in vivo* settings (52, 53). To evaluate whether KDM4 inhibition increased cellular susceptibility to support HBV replication we measured core antigen (HBcAg) by immunofluorescence and showed an increased frequency of antigen-expressing cells following QC6352 treatment (Fig. 3, *F* and *G*). Three patterns of HBcAg distribution were observed: diffuse cytoplasmic staining (1), high-intensity nuclear staining (2), and a uniform distribution throughout the cell (3). Notably, these three staining patterns are seen in human liver biopsy samples from subjects with chronic hepatitis B (CHB), where HBcAg localization is heterogeneous (52). Whilst QC6352 treatment increased the intensity of the HBcAg staining, there were no appreciable changes in the staining pattern, and nuclear staining was the most abundant pattern, irrespective of drug treatment. Collectively, these data show that inhibiting KDM4 enhances the abundance of viral transcripts from both integrated and extra-chromosomal HBV genomes and subsequent HBcAg expression.

### KDM4 regulates hepatocyte susceptibility to support HBV replication

**Figure 4:**
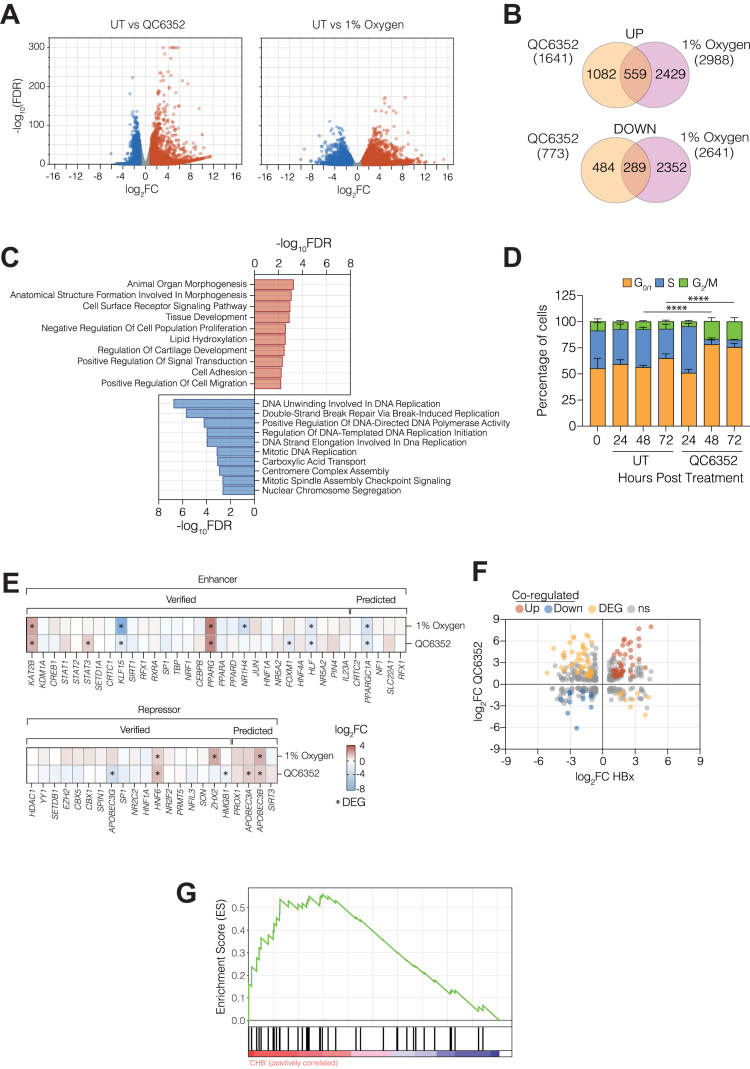
**KDM4 and HBx co-regulate hepatocyte gene expression and cell cycle.***A*, differential expression analysis (DESeq2) of host genes in QC6352 (5 μM) treated or hypoxic (1% oxygen) cultured HepG2-NTCP cells (log_2_FC > ±1 and FDR < 0.05). Up-regulated genes are shown in *red* and down-regulated genes are shown in *blue*. *B*, Venn diagrams showing the overlap of up- and downregulated genes in response to QC6352 treatment or hypoxic (1% oxygen) culture. *C*, pathway analysis of the up- (*red*) and down- (*blue*) regulated genes co-regulated by both hypoxic culture and QC6352 treatment from (*B*). Pathway analysis was performed using Gene Ontology terms and PantherDB. Sequencing data were obtained from three independent biological replicates. *D*, QC6352 (5 μM) or untreated HepG2-NTCP cells were sampled every 24 h, and the cell cycle phase was measured by quantifying BrdU incorporation and propidium iodide staining. Differences in S-phase populations were assessed (2-way ANOVA ∗∗∗∗*p* < 0.0001). *E*, effect of QC6352 treatment or hypoxia (1% oxygen) on the expression of host genes whose proteins interact with HBV cccDNA, where ∗denotes differentially expressed genes based on thresholds for significance of log_2_FC ± 1 and FDR < 0.05. *F*, scatter plot showing QC6352 and HBx regulated gene expression (log_2_FC) where co-upregulated genes (*red*) or down-regulated genes (*blue*) are shown. *Orange points* represent genes that were differentially regulated by either HBx or QC6352 treatment. *G*, GSEA of the 48 HBx and QC6352 co-regulated genes shows increased expression (Normalised Enrichment Score = 1.56; FDR = 0.014) in CHB liver (n = 90) compared to non-viral infected controls (n = 6). Data are presented as mean ± SD from (*D*) n = 6, and significance is assessed by 2-way ANOVA (∗*p* < 0.05, ∗∗*p* < 0.01, ∗∗∗*p* < 0.001, ∗∗∗∗*p* < 0.0001). See Fig. S5.

As KDM4 specifically regulates repressive methylation marks on H3K9, and our data showed an absence of this mark on the viral genome, we were interested in understanding the impact of its inhibition on hepatocellular gene expression. Batie and Chakraborty (28, 29) identified KDM5 and 6 as major transcriptional regulators *via* chromatin modification. Therefore, it is likely that a complex interplay between KDMs, HIFs, and other 2-OGDDs regulate the cellular hypoxic response. While HBV cccDNA associates with histones and is chromatinized (38, 54), we hypothesized that KDM4 regulates the expression of host genes essential for HBV transcription and replication. Analyzing the host transcriptome of QC6352-treated HepG2 cells revealed 1641 up-regulated and 773 down-regulated protein-coding genes (Fig. 4*A*) (55). Of these, one-third of transcripts were differentially expressed in response to hypoxic culture (1% oxygen), consistent with the oxygen-dependent activity of KDM4 (Fig. 4*B*).

Hepatocytes are instrumental in the metabolism and detoxification of drugs, and this is largely a result of Cytochrome P450 (CYP) expression and activity (reviewed in (56, 57)). *Ex vivo* culture of hepatocytes can result in a loss of the enzymatic activity of these factors, but interestingly we observed an upregulation of CYP3A4, CYP1A2, CYP2D6, and CYP2D8, among the differentially expressed genes in response to QC6352 treatment (58, 59). Pathway analysis of QC6352 treated cells showed an over-representation of genes involved in cell cycle regulation and DNA damage response pathways amongst the downregulated transcripts (Fig. 4*C*). We noted an enrichment of up-regulated genes associated with morphogenesis and developmental processes (Fig. 4*C*). These data are consistent with reports identifying a role for KDM4 in organ formation, regulation of stem cell potency and early embryonic development (reviewed in (60)), reflecting the major role that 2-OGDDs play in regulating the host cell response to hypoxic and metabolic stimuli.

Hepatocytes in the liver are typically in the G_0_ phase of the cell cycle and non-proliferating. Mitotic cell cycle arrest has been implicated in maintaining the cccDNA reservoir and cell division *in vitro* has been associated with a loss or dilution of cccDNA (61, 62). Hence many infection protocols culture primary hepatocytes and hepatoma lines with 1 to 2.5% DMSO to arrest cells. Since QC6352 is reported to have antiproliferative properties in the context of breast cancer (36), and our pathway analysis highlighted cell cycle dysregulation, we measured the effect of QC6352 on the HepG2 cell cycle. Bromodeoxyuridine (BrdU) incorporation showed that 48 h of treatment increased the frequency of cells in G_0/1_ and G_2_/M phases, with very few cells in S-phase, and this phenotype persisted for the duration of the experiment (Fig. 4*D*).

Our data show that KDM4 inhibition provides a pro-viral environment that supports HBV replication. To explore the underlying mechanism, we assessed whether KDM4 altered the gene expression of host factors reported to bind cccDNA. Analyzing the transcriptome of HepG2-NTCP cells treated with QC6352 or cultured under 1% oxygen for 72 h, revealed that QC6352 altered the expression of several genes reported to enhance or repress HBV replication (63). Notably, we observed an upregulation of Peroxisome Proliferator-Activator Receptor (PPAR) gamma, previously reported to positively regulate cccDNA activity through interactions with transcription factors such as C/EBP, RXR and FXR (64). KAT2B, a histone acetyl-transferase that is recruited by HBx to activate cccDNA transcription, was increased in QC6352 treated or hypoxic cells (Figs. 4*E* and S5*A*) (65). High mobility group box 1 (HMGB1), a recently identified HBV restriction factor (12), was downregulated by QC6352 treatment but not hypoxic conditions, consistent with a role for multiple oxygen-sensitive pathways in defining cellular permissivity to support HBV replication (Fig. 4*E*).

HBx positively regulates viral transcription *via* multiple pathways, including perturbation of host repressor and activator pathways (reviewed in (1, 66)). Since KDM4 has pleiotropic effects on the host transcriptome, we sought to assess the potential interplay between HBx and KDM4-regulated gene expression and associated pathways. A recent study reported the cellular transcriptional response to HBx (67) and analyzing these data along with our QC6352 RNA-seq data identified several co-regulated genes (Fig. 4*F*). 37 genes were upregulated (shown in red) and 11 genes (shown in blue) were downregulated by both QC6352 and HBx (Table S1). None of these genes encoded proteins that were previously reported to be part of the cccDNA interactome or to influence HBV replication under normoxic conditions (63). Furthermore, gene ontology analysis revealed they were not linked to any common biological processes or pathways. We previously reported increased hypoxic gene expression in the CHB liver (68) and were interested in analyzing these published transcriptomic data sets for perturbation of KDM4 and HBx co-regulated genes. Gene set enrichment analysis (GSEA) of the co-regulated genes identified an increased expression (Normalized Enrichment Score = 1.56; FDR = 0.014) in the liver tissue from CHB patients (Fig. 4*G*). Of note, there were no detectable changes in KDM4A-D transcripts in CHB liver, however, our results support a perturbation of KDM4-regulated gene expression in the diseased liver (Fig. S5*B*). Collectively, these data show a role for KDM4 to regulate several pathways that may determine host susceptibility to HBV infection. Our observation that HBx and KDM4 share transcriptional target genes suggests that oxygen-sensing mechanisms and HBx have a functional overlap to foster a permissive environment for HBV to initiate early infection events.

## Discussion

Hepatocyte function is regulated by the natural oxygen gradient in the liver, and our earlier study identified a role for hypoxic conditions to potentiate viral replication *via* HIF binding and activation of HBV cccDNA transcription (16). Our work indicates that HIFs are not the only oxygen-sensitive elements that regulate HBV, and we identified KDM4 as a novel host restriction factor for the virus. Our transcriptomic studies showed that QC6352 treatment significantly altered hepatocellular gene expression and host cell processes, which are likely to promote viral replication. We showed a limited role for KDM4 to regulate expression of known cccDNA activators or repressors (63), but found that KDM4 and HBx share transcriptional targets. Comparing HBx transcriptomic data (67) identified 48 genes that were co-regulated by KDM4 and further analysis of published HBx ChIP-seq data showed minimal evidence for HBx binding to any of the 48 gene promoters, consistent with an indirect role for HBx in regulating host gene expression (69). These genes were diverse and not over represented in any defined biological pathway or process, but were enriched in the CHB liver, consistent with our earlier study demonstrating increased hypoxic gene expression (68, 70). Interestingly these genes were enriched in liver samples from a chronic HCV cohort, suggesting common hypoxic and inflammatory pathways in the diseased liver. These data are consistent with a model where hepatocytes in low oxygen or hypoxic areas of the liver are more permissive to HBV infection and inhibition of KDM4 may act in concert with HBx to promote viral transcription. In support of this, we previously reported an association between HIF-signaling and HBcAg expression in the HBV-transgenic mouse model, where pericentral localization of HBcAg-expressing cells was reduced in mice following HIF-1β silencing (16).

*De novo* HBV-infected cells were the most responsive to KDM4 inhibition and showed the greatest induction of viral RNAs. Despite the transcript fragmentation used in our Illumina RNA-seq experiments, we noted an enrichment of viral reads spanning the HBs encoding region of the genome consistent with our recent long-read sequencing analysis (48). HBV-infected HepG2 cells will largely transcribe from cccDNA genomes, and epigenetic variability such as DNA, RNA, and histone modifications between model systems may define their sensitivity to KDM4 and hypoxic regulation. For example, a study from Liu *et al.* (71) showed that integrated HBV genomes in Huh-1 cells were highly methylated (5-methylcytosine), whereas cccDNA was unmethylated. The differential sensitivity between viral integrant cell lines may be attributed to the integration site within the host genome that may be less accessible to host replication complexes. Viral integrants may be influenced by the epigenetic status of nearby genes. For instance, it has been demonstrated that HBV integrates in regions with a high concentration of CpG islands, which may affect responses to epigenetic modifiers (50). Response to HIF mimetics and 2-OGDD inhibitors may be dynamic and cell type specific, as a recent study from Gilmore *et al.* (72) found that siRNA silencing of KDM4A-E had a modest effect on the level of HBV RNAs and antigens in experimentally infected human hepatocytes. The differences between our studies may reflect the experimental design, where Gilmore *et al.* assessed the effects of siRNA 14 days post-infection and showed that silencing KDM5 reduced the level of HBV RNAs. Overall, there is likely to be an interplay between KDMs, HIFs and other 2-OGDDs in the hypoxic environment of the liver, and the sequence of events and mechanisms defining the oxygen-dependent regulation of HBV will be dynamic.

The wide-ranging effects of hypoxia on host gene expression are reflected in our transcriptomic analysis, where we see a partial overlap of DEGs between QC6352 treatment and hypoxic (1% oxygen) culture. Low oxygen conditions perturbed twice as many transcripts as QC6352 treatment, suggesting that inhibition of other 2-OGDDs together with stabilization of HIFs have a greater impact on host gene expression than KDM4 inhibition alone. The KDM superfamily members have varying degrees of sensitivity to oxygen, with reported Michaelis constants for molecular oxygen, K_m_O_2_, ranging between 20 to 250 mM (73). Selective inhibition of specific 2-OGDDs with hypoxic culture is not possible and significant effort has been invested in the development of pharmacological agents that are selective. Of note, QC6352 was reported to be a highly selective KDM4 inhibitor (IC_50_ 34–104 nM) (36) and CPI-455 has specificity for KDM5 (IC_50_ 10 nM) (32). In contrast, GSK-J4 can modulate both H3K4me3 and H3K27me3, suggesting cross-reactivity with both KDM5 and KDM6 enzymes despite a high affinity for KDM6 (IC_50_ 60 nM) (33, 74). Pharmacological inhibition of any 2-OGDD in an otherwise normoxic cell is likely to oversimplify the extent of the cellular consequences, as hypoxia will result in dynamic changes to several histone modifications (28, 29, 75).

KDMs may influence cellular susceptibility to support infection by other viruses, particularly large DNA viruses that associate with host chromatin. The Epstein-Barr virus genome preferentially associates with H3K9me3-rich areas of the host nucleosome in B-lymphocytes, implicating KDM4 in the persistence of this pathogen (76). Similarly, associations between H3K9me3 and the late gene-coding region of HPV 16 in keratinocytes show potential avenues for KDM4-mediated modification (76). Furthermore, our data suggest that local oxygen tension could define the efficacy of epigenetic modifying agents such as the histone deacetylase (HDAC) inhibitors currently being investigated as therapies for HPV and HIV (77). Our data highlight the importance of developing model systems that incorporate local oxygen tension when screening antiviral agents and this is relevant for the evaluation of anti-cancer and immune therapies. Given the wide range of host genes and pathways we found to be regulated by KDM4, it would not be surprising if QC6352 treatment affected infection with other hepatotropic viruses, such as hepatitis C or D viruses. Beyond virology, HBV cccDNA resembles extra-chromosomal DNA (ecDNA) that is implicated in carcinogenesis and can promote oncogene expression (78, 79). Oxygen-sensitive chromatin modulators, such as KDMs, may play a role in regulating ecDNA transcription.

H3K9me3 is widely recognized as the major target for KDM4 (80) and we opted to use this as a surrogate for KDM4 inhibition throughout our study. However, there is increasing literature on purported histone and non-histone substrates for KDM4. Notably, H3K36me3 is a mark associated with transcriptional activation, and is considered to be a substrate for KDM4 but reported to be absent on cccDNA molecules in *in vitro* and *in vivo* samples (38). However, due to the simultaneous inhibition of 2-OGDDs and potential cross-reactivity of pharmacological agents, it is difficult to resolve the cognate specificity of enzyme and substrate. Biochemical assays have been used to identify KDM4:substrate binding as well as demethylase activity. H3K9me2, H3K36me2/3, H1.4K26me2/3, H3K56me3 and even H3K27me2/3 are purportedly substrates for KDM4 mediated demethylation (81, 82, 83, 84). Identifying enzyme-specific substrates remains challenging, as cell-free assays and overexpression studies may not reflect endogenous protein expression, intracellular location or native binding partner availability (85). It is important to acknowledge that alternative tri- and di-methylated histone post-translational modifications could regulate gene expression from either cccDNA or viral integrants (38), and other KDMs are likely to be involved in the viral life cycle.

HBV is considered a stealth virus and earlier studies identified modest changes in the bulk transcriptome of infected cells *in vitro* and using experimental mouse models or liver biopsies from CHB patients (43, 70, 86, 87). The hepatic transcriptome is influenced by liver oxygenation, and the overlap in HBx and KDM4-regulated genes highlights a fundamental role for KDM4 to regulate host pathways key for viral replication (14). Of note, we observed that QC6352 treatment increased the expression of several cytochrome transcripts that are considered markers of hepatocyte function in drug detoxification (88). We found that CYP3A4 was KDM4 regulated, and has been shown to be enriched in the pericentral region of the human liver, consistent with oxygen-driven liver zonation (89). Interestingly, *in vitro* assays have manipulated cytochrome expression to regulate the differentiation of hepatocyte-like cells from induced pluripotent stem cells (44, 46, 58). Our data suggest that QC6352 may influence the hepatocyte-like status of HepG2-NTCP cells and warrants further investigation. We acknowledge the limitations of our HBx transcriptomic analysis, which was derived from a plasmid-based overexpression system in HepG2 cells (67) that may differ from the authentic viral transcriptome. However, our demonstration of increased KDM4-HBx co-regulated gene expression in CHB liver biopsies provides evidence that these pathways are active in the diseased liver. We recently identified a 24-inflammatory gene signature that is associated with cccDNA transcriptional activity in CHB, which was attributed to Kupffer and liver endothelial cells (90). These data support a model in which oxygen signaling pathways in non-parenchymal cells may influence HBV replication through the release of cytokines and signaling molecules.

QC6352 has been used as an antiproliferative agent in mice harboring breast cancer tumor xenografts, as well as anti-cancer activity in both rhabdomyosarcoma and squamous cell carcinoma, and our data shows that KDM4 arrests HepG2 cell cycle most likely by perturbing mitotic spindle formation and dysregulating the DNA damage response (36, 91, 92). This appears to be characteristic of KDM4 inhibitors, as QC8222 has entered a trial for advanced metastatic cancers (93), and the pan-KDM inhibitor JIB-04 is currently in clinical trial against breast leptomeningeal carcinomatosis, due to KDM4A and C inhibition (94). As the majority of *in vitro* studies investigating HBV replication have been performed at 18% oxygen, where KDM4 is active, this may explain the low levels of HBV replication compared with experimental animal models (42). As KDM4A-D isoforms are expressed in hepatocytes and all hepatoma cell lines tested to date, their ongoing demethylase activity under standard laboratory culture conditions may inhibit HBV replication *in vitro*. Culturing hepatoma lines with DMSO is commonly used to arrest their proliferation and to promote the expression of hepatocyte-specific transcription factors (61). Our earlier study showed that DMSO blunts the cellular response to hypoxia and dampens the induction of canonical HIF-regulated genes (16). Hence the majority of experiments in this study were performed in the absence of DMSO which allowed us to identify a role for QC6352 treatment to arrest HepG2 cells and to promote the expression of hepatocyte-specific factors that is worthy of further investigation. Our study highlights a role for KDM4 to restrict HBV replication and pharmacological inhibition of KDM4 increases the steady-state levels of HBV RNA and HBcAg, arrests cell cycle progression, and provides a valuable model for studying HBV replication. Elucidating the oxygen-sensitive pathways that are activated or repressed in the diseased liver may lead to future studies aimed at uncovering and validating druggable targets.

## Experimental procedures

### Cells and reagents

HepG2-NTCP, HepAD38, Huh1, PLC/PRF5, and Hep3B cells were maintained in DMEM (ThermoFisher) containing GlutaMAX plus 10% FBS, 50 U/ml Penicillin/Streptomycin, and non-essential amino acids. Plasticware was pre-treated with collagen (Sigma) and maintained at 37 °C with 5% CO_2_/18% oxygen. Hypoxic treatments were performed in a hypoxic chamber (3%, or 1% oxygen) (Invivo 400, Baker-Ruskinn Technologies). JIB-04, QC6352, CPI-455 and GSK-J4 were purchased from MedChem Express.

### HBV infection

HBV was purified from HepAD38 cells using heparin-affinity chromatography as previously reported (95). HepG2-NTCP cells were infected with HBV MOI 300 with 4% polyethylene glycol-8000, washed after 6 h and treatments applied.

### siRNA transfections

HepG2-NTCP cells were transfected with a 50 nM pool of siRNAs targeting KDM4A-D in an equimolar ratio (96), or scrambled control (Ambion) with DharmaFECT (Dharmacon). Transfected cells were left to recover for 4 h, before changing the medium and infection with HBV.

### Cell cycle FACS

Cells were incubated with 10 μM bromodeoxyuridine for 60 min at 37 °C, trypsinized and washed with PBS before fixing in ice cold 70% ethanol. Cells were incubated with pepsin (1 mg/ml, 37 °C), treated with 2 M HCl for 15 min and washed before blocking with 0.5% BSA/0.5% Tween for 30 min. Cells were stained with anti-BrdU-488 (BioLegend) and propidium iodide (Nalgene) for 30 min and analyzed (CyAn flow cytometer, BD Biosciences).

### RT-qPCR and qPCR

Cellular RNA or DNA were extracted using RNeasy or AllPrep (Qiagen) kits respectively, using on-column DNAse treatment to remove viral DNA carry-over. RNA concentration was measured using a Nanodrop-1000 (Thermo Fisher), and 1 μg was reverse-transcribed with a random hexamer/Oligo-dT cDNA synthesis kit (PCRBIO). Gene expression was analyzed using the conventional ΔΔCt method relative to either β-Actin or RPLP0 housekeeping genes. HBV cccDNA or HBV genomes were enumerated as previously described (34). cccDNA was quantified by qPCR of DNA treated with T5 exonuclease (NEB, UK) and expressed relative to the host PrP gene. All qPCRs were performed using a 2-step SYBR green Mastermix (PCRBIO) with the oligonucleotides listed in Table S2.

### Quantification of extracellular HBV rcDNA

To quantify extracellular HBV rcDNA, supernatants were treated with 10 U of DNase (ThermoFisher) at 37 °C for 30 min. DNase was inactivated with 1 mM EDTA, before adding 2× lysis buffer (0.1 M Tris-HCl (pH7.4), 50 mM KCl, 0.25% Triton X-100, 40% glycerol). HBV DNA was enumerated using HBV total DNA primers (38), against a DNA reference standard curve.

### HBV antigen ELISAs

HBeAg was detected in the supernatant of HepG2-NTCP cells infected with HBV according to the manufacturer’s instructions (Autobio).

### SDS-PAGE and Western blot

Samples were lysed in RIPA buffer (20 mM Tris, pH 7.5, 2 mM EDTA, 150 mM NaCl, 1% NP40, 1% sodium deoxycholate, 1% sodium dodecyl sulphate) supplemented with protease inhibitors (Roche). Proteins were detected using primary and HRP-conjugated secondary antibodies using the SuperSignal West Pico kit (Pierce) visualized on a G:Box mini (Syngene). The following primary antibodies were used: KDM4A (Abcam), β-actin (Sigma), HIF-1α (BD Bioscience), H3 (CST), H3K4me3 (Active Motif), H3K9me3 (Sigma), H3K27me3 (Active Motif) and NDRG1 (CST). All bands were compared against a molecular weight ladder (NEB) and the antibody data sheet, to confirm product-specific detection. Figures presented show all detected bands.

### Chromatin immuno-precipitation

As previously reported (16), HepAD38 cells were cultured on collagen-coated 100 mm dishes in the presence or absence of QC6352 (5 μM) for 72 h. Cultures were fixed in 1% formaldehyde (Sigma) for 10 min before quenching in 125 mM glycine. Cells were washed twice in ice-cold PBS, before scraping and pelleting (800 rpm, 10 min), then lysed in 500 μl of nuclear extraction buffer (10 mM Tris-HCl (pH 8), 10 mM NaCl, 1% NP-40, supplemented with protease inhibitor (Roche)). Chromatin was diluted 1:1 (0.01% SDS, 1.1% Triton, 0.2 mM EDTA, 16.7 mM Tris (pH 8.1), 167 mM NaCl) and sheared by sonication (Bioruptor, high power 30 min 15 s on/off). After centrifugation (1300 rpm, 10 min) lysates were immunoprecipitated overnight with 2 to 5 μg of antibodies specific to H3K4me3 (Active Motif), H3K9me3 (Active Motif), H3K27me3 (Active Motif) or an irrelevant IgG control (Sigma) then pulled down with protein agarose beads (Millipore, 16–156). Immuno-precipitates were washed in low salt buffer (0.1% SDS, 1% Triton, 2 mM EDTA, 20 mM Tris pH 8.1, 150 mM NaCl), high salt buffer (0.1% SDS, 1% Triton, 2 mM EDTA, 20 mM Tris pH 8.1, 500 mM NaCl), lithium chloride buffer (1% Igepal, 1 mM EDTA, 10 mM Tris pH 8.1, 250 mM LiCl, 1% sodium deoxycholate) and finally twice in TE buffer (0.1 M Tris pH 8, 1 mM EDTA), before elution in 240 μl of elution buffer (0.1 M NaHCO_3_, 1% SDS). Reverse crosslinking was carried out at 65 °C overnight, in the presence of 200 mM NaCl, prior to digestion with Proteinase K and RNaseA, before on-column cleanup (Qiagen MiniElute PCR purification) as per the manufacturers’ instruction.

### ChIP RT-qPCR

Samples were amplified using a SYBR green RT-qPCR (PCR Biosystems) using primers specific for either HBV regulatory loci as defined by Tropberger *et al.* (38), or host gene loci, with sequences listed in Table S2. Control genes were selected from previous reports, as either high (+) or low (−) association with specific histone marks. GAPDH: H3K4me3+, H3K9me3-, H3K27me3- (41); Sat2B: H3K4me3-, H3K27me3+ (40); ZNF510: H3K9me3+ (39); TSH2B: H3K27me3+ (39). Percentage of input was calculated for both treated and untreated cells and expressed relative to the IgG control.

### HBcAg immuno-staining

HepG2-NTCP cells were cultured on cover slips in the presence of 2.5% DMSO for 3 days, and then infected with HBV (MOI 1000). Immediately post-infection cells were treated with 5 μM QC6352, which was replenished every 3 days, before fixation in 4% PFA at 10 days post-infection. Cells were permeabilized (0.1% Triton-X100, 30 min), blocked (3% BSA, 30 min), then stained with rabbit anti-HBc antibody (Dako) for 1 h and a 488-conjugated anti-rabbit secondary antibody (Thermofisher) for 40 min. Coverslips were incubated in DAPI for 10 min before washing and mounting using Prolong Anti-fade (LifeTechnologies). Samples were imaged on an Olympus SoRa super-resolution confocal microscope. Quantification was performed using ImageJ software (Fiji). The number of infected cells and average intensity of core protein for both conditions were quantified with Fiji open-source software. The average intensity of infected cells was measured within the infected area, which was defined based on an automatic threshold with a Minimum Error algorithm. The number of cells was quantified based on the DAPI signal. Briefly, the image was blurred using a Gaussian filter (sigma = 4), and the threshold applied using Otsu algorithm. A watershed step was performed to separate nuclei in close proximity. Infected cells were defined based on an HBcAg signal intensity threshold of at least 180. Each field of view contained approximately 400 cells, and ten fields of view were imaged per condition.

### RNA-seq analysis

HepG2-NTCP cells were infected with HBV Genotype D strain ayw (MOI 300) or mock for 6 h, washed and treated with QC6352 (5 μM), FG-4592 (30 μM) or cultured at 18% or 1% oxygen for 72 h and harvested for RNA extraction. RNA integrity was measured by Tapestation (Agilent), before polyA-enriched transcriptome sequencing (Novogene). Paired-end Illumina sequencing (300 bp) was mapped to the human genome using STAR 2.7 and viral reads were mapped to the HBV genome, where the start coordinate is the pgRNA TSS (nt1816) (7). TPM values and differential expression were quantified (DESeq2 package). The threshold for statistical significance was set at log_2_FC > ±1 and FDR <0.05.

### GSEA

Analysis was carried out using GSEA_4.1.0 and performed using phenotype grouping between CHB patients and non-viral subjects.

### Statistical analysis

All analyses were carried out in Prism 9. Data are shown as means ± SD with probabilities represented by ∗*p* <0.05, ∗∗*p* <0.01, ∗∗∗*p* <0.001 and ∗∗∗∗*p* <0.0001. Where appropriate, *p* values were corrected for multiple comparisons by the Bonferroni method (Mann Whitney U tests) or the original Benjamini-Hochberg method (for FDR assessment).

## Data availability

RNA-seq data are available at Gene Expression Omnibus (GSE186279 and GSE186178) (55). Published RNA-seq data from HepG2 cells, cultured at 0.5% oxygen for 16 h (GSE120886) (97). The CHB microarray data is available as GSE83148 (70). Primary data are available through Mendeley Data: Harris, James (2024), ‘Oxygen-dependent histone lysine demethylase 4 restricts hepatitis B virus replication’, Mendeley Data, V1, https://doi.org/10.17632/m6j6jzzt36.1.

## Supporting information

The article contains supporting information (38).

## Conflict of interest

The authors declare that they have no conflicts of interest with the contents of this article.

## Supporting information

Supporting Figure S1 **Silencing KDM4.***A*, HepG2-NTCP cells were treated with agents targeting KDM4, KDM5, KDM6 or the pan-KDM inhibitor JIB-04, as described in Figure 1. Cells were lysed and histone modifications were assessed by western blotting. *B*, HepG2-NTCP cells were treated with QC6352 in a serial dilution from 9 μM to 0.12 μM. Cell viability was assessed using an LDH assay and expressed relative to UT cells. Data were obtained from two independent experiments, each consisting of at least four biological replicates. *C*, HepAD38 cells were cultured in the presence of QC6352 (5 μM) for 72 h. Cells were fixed, and chromatin extracted before immunoprecipitation with antibodies specific to H3K4me3, H3K27me3 or an irrelevant IgG control. RT-qPCR was used to amplify either HBV DNA (38) or host gene loci, and percentage of input was calculated. Data are expressed as fold above the IgG control in each condition for each primer pair, and are derived from two independent experiments. *D*, expression heatmap of KDM4 isoforms in hepatoma cells and primary hepatocytes from published RNA-sequencing datasets. KDM4A-D normalised read-counts are expressed relative to the geometric mean of β-Actin and RPLP0 housekeeping gene reads. *E*, expression of KDM4 isoforms in response to transient silencing of KDM4A-D in HepG2-NTCP cells, 72 h post siRNA delivery. Data are representative of 2 independent experiments, and statistics tested with Mann-Whitney U tests (∗*p* < 0.05). **Supporting Figure S2**: **Hypoxic gene expression in HepG2-NTCP cells.***A*, HepG2-NTCP cells were cultured in 18%, 3% and 1% oxygen for 72 h. RNA was extracted and expression of previously identified HIF target genes was quantified by qPCR. Data were expressed relative to normoxic cultured cells, and statistical significance was assessed using independent Mann-Whitney U tests. *B*, HepG2-NTCP cells were treated with QC6352 (5 μM) for 72 h, and HIF target gene transcripts were assessed by qPCR. Data are expressed relative to untreated cells, represented by the dashed line. Statistical significance was determined by Mann-Whitney U tests (∗*p* < 0.05, ∗∗*p* < 0.01). **Supporting Figure S3**: **QC6352 activity under hypoxic conditions. (a)** HBV-infected HepG2-NTCP cells were cultured at 18% or 1% oxygen ± QC6352 (5 μM) for 72 h and pC/pgRNA copies determined by qPCR. Data are presented relative to untreated cells at each oxygen concentration. Statistical analyses were performed using independent Mann-Whitney U tests (∗*p* < 0.05). **Supporting Figure S4**: QC6352 promotes pC/pgRNA levels in HepAD38 cells (*A*) HepAD38 cells were treated with QC6352 (5 μM) for 72 h and intracellular pC/pgRNA copies determined by qPCR. Data were derived from two independent experiments, with data presented relative to untreated cells. Statistical analysis was performed using the Mann-Whitney U test (∗*p* < 0.05). **Supporting Figure S5**: KDM4 expression in CHB **(a)** HepG2-NTCP cells were treated with 5 μM QC6352 for 72 h and expression of PPARG and KAT2B determined by qPCR. Data are expressed relative to untreated cells and statistics tested with Mann-Whitney U tests (∗∗*p* < 0.01). *B*, expression of KDM4 isoforms in CHB (n = 90) patients and non-viral controls (n = 6). Intrahepatic gene expression was quantified using Affymetrix microarray on RNA extracted from liver biopsies. Normalised expression values were plotted, and statistics were tested using the Mann-Whitney U test. **Supporting Table S1**: HBx and KDM4 co-regulated genes, as described in Figure 4f. **Supporting Table S2**: Nucleotide sequences of qPCR primers (5′-3′) used in this study.
