# Supplementary material for: Oxygen-dependent histone lysine demethylase 4 restricts hepatitis B virus replication
Source: J Biol Chem. 2024 Feb 5;300(3):105724. doi: 10.1016/j.jbc.2024.105724 (PMC10914488; doi:10.1016/j.jbc.2024.105724)
Supplement: Supporting Figure S1 — Silencing KDM4.A, HepG2-NTCP cells were treated with agents targeting KDM4, KDM5, KDM6 or the pan-KDM inhibitor JIB-04, as described in Figure 1. Cells were lysed and histone modifications were assessed by western blotting. B, HepG2-NTCP cells were treated with QC6352 in a serial dilution from 9 μM to 0.12 μM. Cell viability was assessed using an LDH assay and expressed relative to UT cells. Data were obtained from two independent experiments, each consisting of at least four biological replicates. C, HepAD38 cells were cultured in the presence of QC6352 (5 μM) for 72 h. Cells were fixed, and chromatin extracted before immunoprecipitation with antibodies specific to H3K4me3, H3K27me3 or an irrelevant IgG control. RT-qPCR was used to amplify either HBV DNA (38) or host gene loci, and percentage of input was calculated. Data are expressed as fold above the IgG control in each condition for each primer pair, and are derived from two independent experiments. D, expression heatmap of KDM4 isoforms in hepatoma cells and primary hepatocytes from published RNA-sequencing datasets. KDM4A-D normalised read-counts are expressed relative to the geometric mean of β-Actin and RPLP0 housekeeping gene reads. E, expression of KDM4 isoforms in response to transient silencing of KDM4A-D in HepG2-NTCP cells, 72 h post siRNA delivery. Data are representative of 2 independent experiments, and statistics tested with Mann-Whitney U tests (∗p < 0.05). Supporting Figure S2: Hypoxic gene expression in HepG2-NTCP cells.A, HepG2-NTCP cells were cultured in 18%, 3% and 1% oxygen for 72 h. RNA was extracted and expression of previously identified HIF target genes was quantified by qPCR. Data were expressed relative to normoxic cultured cells, and statistical significance was assessed using independent Mann-Whitney U tests. B, HepG2-NTCP cells were treated with QC6352 (5 μM) for 72 h, and HIF target gene transcripts were assessed by qPCR. Data are expressed relative to untreated cells, represented [file mmc1.pdf]

Oxygen-dependent histone lysine demethylase 4 restricts hepatitis B virus replication.

James M Harris, Andrea Magri, Ana Rita Faria, Senko Tsukuda, Peter Balfe, Peter AC Wing and Jane A McKeating

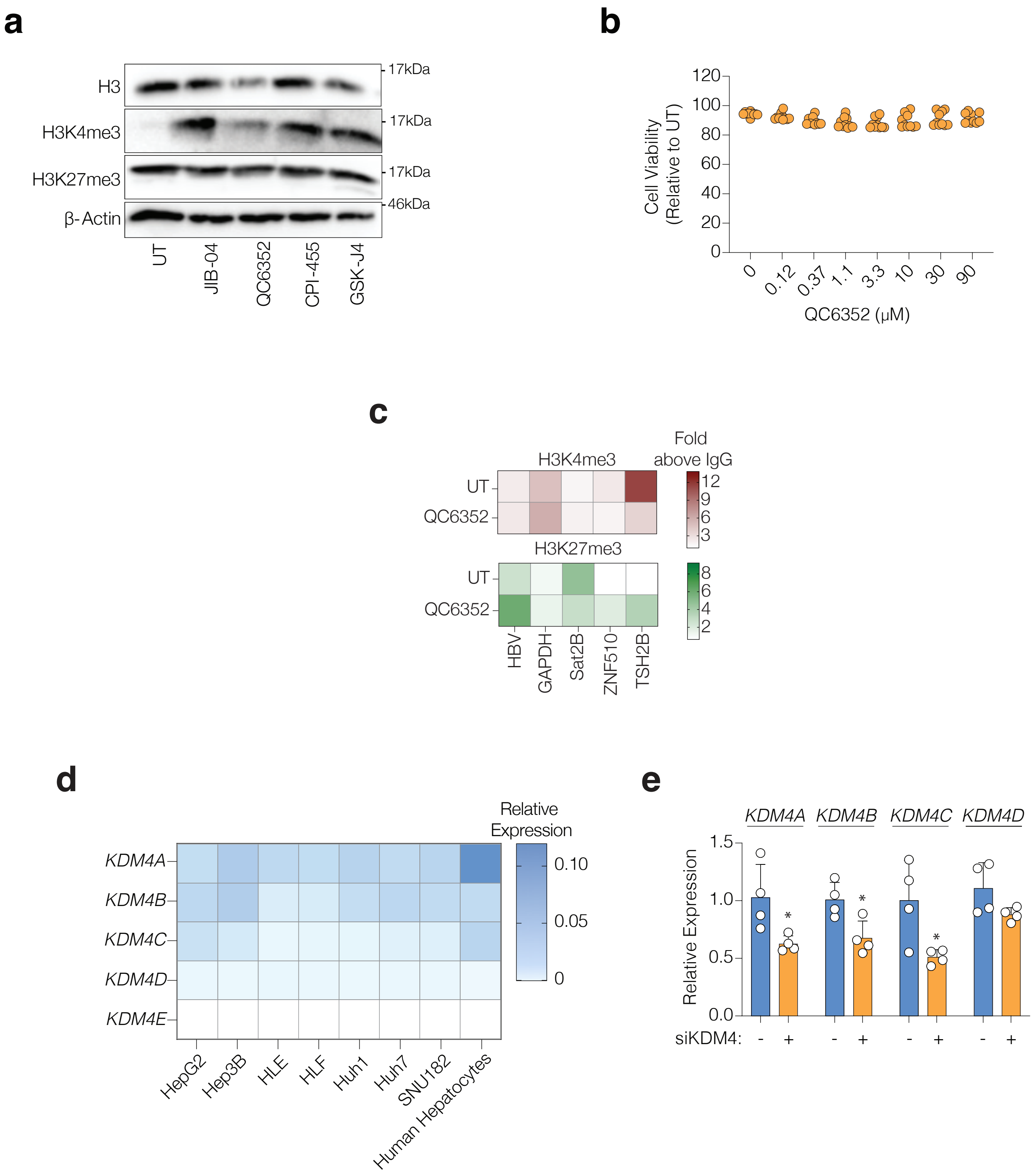

Supplemental Figure 1 - Harris et al.

a

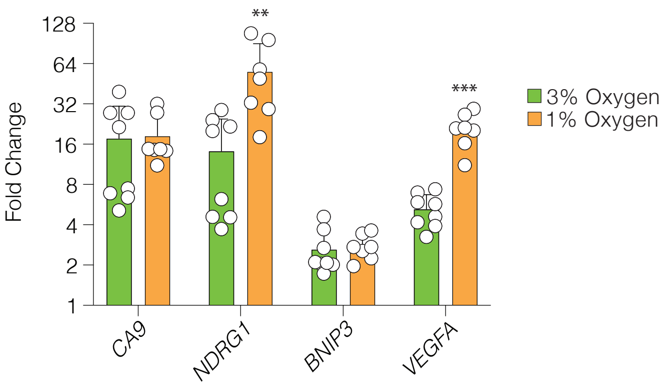

b

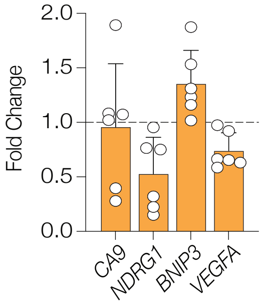

Supplemental Figure 2 - Harris et al.

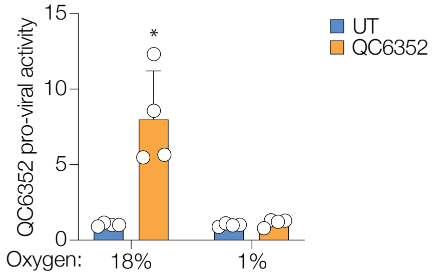

Supplemental Figure 3 - Harris et al.

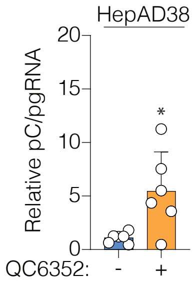

Supplemental Figure 4 - Harris et al.

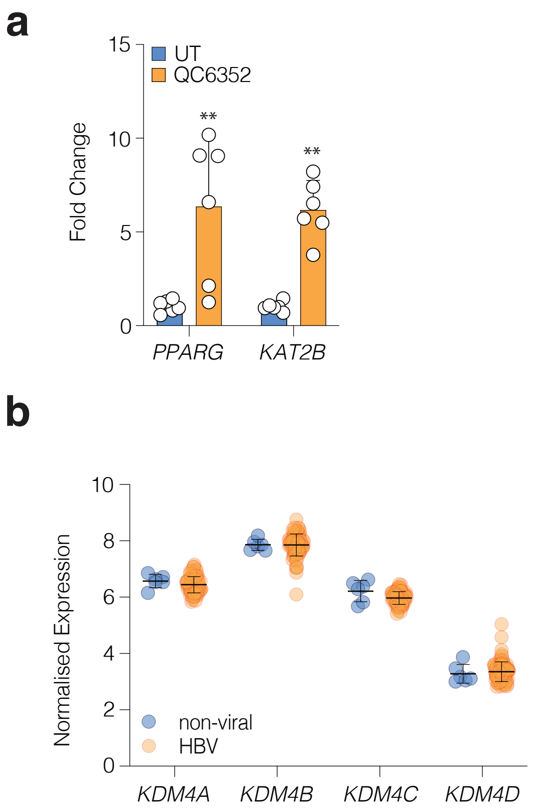

Supplemental Figure 5 - Harris et al.

Up-regulated		Down-regulated	
HIST1H2AG	Histone Cluster 1 H2A Family Member G	DDR2	Discoidin Domain Receptor Tyrosine Kinase 2
TMEM191C	Transmembrane Protein 191C	PDGFRL	Platelet Derived Growth Factor Receptor Like
AARSD1	Alanyl-Trna Synthetase Domain Containing 1	PPP1R27	Protein Phosphatase 1 Regulatory Subunit 27
FAM183A	Family With Sequence Similarity 183 Member A	KCNC3	Potassium Voltage-Gated Channel Subfamily C Member 3
AGAP2	Arfgap With Gtpase Domain, Ankyrin Repeat And Ph Domain 2	LRFN1	Leucine Rich Repeat And Fibronectin Type Iii Domain Containing 1
MT-ATP8	Mitochondrially Encoded Atp Synthase Membrane Subunit 8	APOLD1	Apolipoprotein L Domain Containing 1
FA2H	Fatty Acid 2-Hydroxylase	FAM180B	Family With Sequence Similarity 180 Member B
KNG1	Kininogen 1	REEP1	Receptor Accessory Protein 1
KLHDC7B	Kelch Domain Containing 7B	SLC22A25	Solute Carrier Family 22 Member 25
TMC4	Transmembrane Channel Like 4	CRACR2A	Calcium Release Activated Channel Regulator 2A
KCNK6	Potassium Two Pore Domain Channel Subfamily K Member 6	ARG1	Arginase 1
SCEL	Sciellin		
BSPRY	B-Box And Spry Domain Containing		
TCTEX1D4	Tctex1 Domain Containing 4		
CA2	Carbonic Anhydrase 2		
MSN	Moesin		
ACHE	Acetylcholinesterase (Cartwright Blood Group)		
GSTT2B	Glutathione S-Transferase Theta 2B (Gene/Pseudogene)		
CNBD2	Cyclic Nucleotide Binding Domain Containing 2		
AC099489.1	Vitellogenin		
GEM	Gtp Binding Protein Overexpressed In Skeletal Muscle		
CATSPERG	Cation Channel Sperm Associated Auxiliary Subunit Gamma		
TCN2	Transcobalamin 2		
WNT4	Wnt Family Member 4		
JPH2	Junctophilin 2		
NRG1	Neuregulin 1		
DAO	D-Amino Acid Oxidase		
NODAL	Nodal Growth Differentiation Factor		
DOCK10	Dedicator Of Cytokinesis 10		
EGR2	Early Growth Response 2		
COL17A1	Collagen Type Xvii Alpha 1 Chain		
RAB27B	Rab27B, Member Ras Oncogene Family		
RIMS4	Regulating Synaptic Membrane Exocytosis 4		
SYT1	Synaptotagmin 1		
TNFSF8	Tnf Superfamily Member 8		
CAMK1G	Calcium/Calmodulin Dependent Protein Kinase 1G		
ITGAM	Integrin Subunit Alpha M		

Supplemental Table 1 - Harris et al.

	Forward	Reverse
HBV pC/pg RNA	GGGGAACTAATGACTCTAGCTACC	TTTAGGCCCATATTAGTGTTGACA
HBV preS1/2 RNAs	CTCCAGTTCAGGAACAGTAAACCC	AGGAATCCTGATGTGATGTTCTCC
HBV Total RNAs	ACGGGGCGCACCTCTCTTTA	GTGAAGCGAAGTGCACACGG
HBV cccDNA	GCCTATTGATTGGAAAGTATGT	AGCTGAGGCGGTATCTA
HBV Total DNA	AACACATAGCGCCTCATTTTG	AGGATTGCTGGTGGAAAGATT
ACTB	CCAACCGCGAGAAGATGA	CCAGAGGCGTACAGGGATAG
RPLP0	GCAATGTTGCCAGTGTCTG	GCCTTGACCTTTTCAGCAA
PRP	TGCTGGGAAGTGCCATGAG	GGTGCATGTTTTCACGATAGTA
CA9	CTTGGAAGAAATCGCTGAGG	TGGAAGTAGCGGCTGAAGTC
NDRG1	TTTGATGTCCAGGAGCAGGA	ATGCCGATGTCATGGTAGGT
BNIP3	GCTCCCAGACACCACAAGAT	GAGAGTAGCTGTGCGCTTC
GPI	GGAGCGGATGTTCAATGGT	CGTGATGGTCTTGCCTGT
FUT11	CATCACCAACCAATTTCTTCTG	CATCTCTTTCCAACTGTCATTCTC
P4HA1	GACCACAGCACAGTACAGAG	ATTTGCTACCTGTAATTCCTCTG
KDM4A	AGCTTGCTTAAAGGCTGACG	GAAGTTTCAGTGAGCGGGAG
KDM4B	ATCTTGACCATGTCCTTCCG	TCAACTGCGCAGAATCTACC
KDM4C	CTTTCCTGCAAGTGCACAAT	CCATGCTGGTTTTAATCATGG
KDM4D	GTGGGAGTGAAGAGCACACA	CAGTTACCCAGGAGAGCAGC
PPARG	AGCCTGCGAAAGCCTTTTGGTG	GGCTTCACATTCAGCAAACCTGG
KAT2B	GCACCATCTCAACGAAGACTGC	GTGTGGTTTCGTACCGAGGTAG
HBV1	TTAACAGGCCTATTGATTGGAAA	TCAACGCAGGATAACCACATT
HBV2	AGCAGGCTTTCACTTTCTCG	AACGGGGTAAAGGTTCAGGTA
HBV3	GCATGGACATCGACCCTTA	GGAAAGAAGTCAGAAGGCAAAA
HBV4	AACACATAGCGCCTCATTTTG	AGGATTGCTGGTGGAAAGATT
GAPDH	CGGCTACTAGCGGTTTTACG	AAGAAGATGCGGCTGACTGT
Sat2B	CATCGAATGGAAATGAAAGGAGTC	ACCATTGGATGATTGCAGTCAA
ZNF510	CATCAGTGTCATTCAAGGACG	ACGAGGTTGCTGTAGTTCTCC
TSH2B	CTGACTGAGGTTGGCATTTG	GATTGGACAATGGGAAGTGG
